# Uncovering the multifaceted properties of 6-pentyl-alpha-pyrone for control of plant pathogens

**DOI:** 10.3389/fpls.2024.1420068

**Published:** 2024-06-18

**Authors:** Artemio Mendoza-Mendoza, Edgardo Ulises Esquivel-Naranjo, Sereyboth Soth, Helen Whelan, Hossein Alizadeh, Jesus Francisco Echaide-Aquino, Diwakar Kandula, John G. Hampton

**Affiliations:** ^1^ Faculty of Agriculture and Life Sciences, Lincoln University, Christchurch, New Zealand; ^2^ Unit for Basic and Applied Microbiology, Faculty of Natural Sciences, Autonomous University of Queretaro, Queretaro, Mexico

**Keywords:** 6-pentyl-alpha-pyrone (6-PP), abiotic and biotic interactions, biopesticides, plant pathogens, plant defense regulator, plant growth promotion, sustainability, *Trichoderma*

## Abstract

Some volatile organic compounds (VOCs) produced by microorganisms have the ability to inhibit the growth and development of plant pathogens, induce the activation of plant defenses, and promote plant growth. Among them, 6-pentyl-alpha-pyrone (6-PP), a ketone produced by *Trichoderma* fungi, has emerged as a focal point of interest. 6-PP has been isolated and characterized from thirteen *Trichoderma* species and is the main VOC produced, often accounting for >50% of the total VOCs emitted. This review examines abiotic and biotic interactions regulating the production of 6-PP by *Trichoderma*, and the known effects of 6-PP on plant pathogens through direct and indirect mechanisms including induced systemic resistance. While there are many reports of 6-PP activity against plant pathogens, the vast majority have been from laboratory studies involving only 6-PP and the pathogen, rather than glasshouse or field studies including a host plant in the system. Biopesticides based on 6-PP may well provide an eco-friendly, sustainable management tool for future agricultural production. However, before this can happen, challenges including demonstrating disease control efficacy in the field, developing efficient delivery systems, and determining cost-effective application rates must be overcome before 6-PP’s potential for pathogen control can be turned into reality.

## 1 Introduction

The impact of biotic stress on plant production, caused by attacks from pathogens and pests, has affected humanity’s welfare since the early days of agricultural practices when crops were first cultivated (Howard, 1996). By the beginning of the twentieth century, there was optimism that both diseases and pests would ultimately be controlled with chemicals (Apple, 1977; Grube et al., 2011), but this was not realized, as in the early 1980s James (1981) estimated that global losses due to plant diseases were 12% of potential yield, which was equivalent to a monetary loss of US$50B at the production level. Global yield losses due to diseases and pests are even more prominent in the twenty-first century, ranging from 17% to 47% depending on the crop, season, and location (Glare et al., 2016; Savary et al., 2019; Collinge et al., 2022). Globally, this annual crop yield loss is estimated at US$220B (Singh et al., 2023). Plant diseases, therefore, are exacting a heavy toll on food production with downstream impacts on human health (Savary et al., 2019) and, in some cases, on nations’ social and political stability (Ristaino et al., 2021).

The traditional principles of plant disease control are avoidance, exclusion, eradication, protection, resistance, and therapy (Young, 1968). Avoidance aims to prevent a disease outbreak by selecting a sowing time or site where there is no inoculum or where the environment is not suitable for infection; exclusion aims to prevent the introduction of inoculum; eradication seeks to eliminate, destroy or inactivate the inoculum; protection is used to prevent infection using a toxicant or some other barrier to infection; resistance is the use of resistant germplasm; and therapy is to cure plants already infected (APS, 2024). Integrated disease management (IDM) combines multiple tactics in a strategy to prevent over reliance on any one of these mechanisms (Beckerman et al., 2023); for example, the integration of agrichemicals with cultural practices (site, crop rotation, soil fertility, irrigation), disease-resistant cultivars and possibly also biocontrol agents (BCAs) to achieve sustainable cropping systems. However, for over fifty years, fungicides alone have played a significant role in managing plant diseases (Savary et al., 2019; Beckerman et al., 2023), reducing yield and post-harvest storage losses. Fungicides are currently an essential tool for crop protection, and in the view of Beckerman et al. (2023), they will continue to be an essential component of IDM.

This view contradicts others who seek more sustainable agricultural production systems (Collinge et al., 2022; Pandit et al., 2022; Razo-Belman and Ozuna, 2023). Singh et al. (2023) suggested that fungicide use has reached a plateau. Reasons for this include: i) pathogen resistance to fungicides (Singh and Trivedi, 2017; FRAC, 2020); ii) regulatory requirements (based on toxicity, health risks or environmental persistence) (Beckerman et al., 2023; Singh et al., 2023); iii) consumer demands for food safety, reduced pesticide use and human health impacts (Glare et al., 2016; Singh et al., 2023); iv) a reduction in new synthetic chemistry (Glare et al., 2016); and v) the tremendous cost of producing new fungicides (Bryson and Brix, 2018), which means that because economics prioritizes pathogen and crop targets for new fungicides (Oliver and Beckerman, 2022), any new products developed are only available for the world’s major crops (Beckerman et al., 2023b).

Where conventional approaches to disease control are limited or compromised, biological control offers opportunities (Collinge et al., 2022). Biocontrol methodologies are considered environmentally benign, relatively inexpensive, and can potentially boost plant production significantly (Pandit et al., 2022). Research into biocontrol using antagonistic bacteria, fungi, oomycetes, and viruses has resulted in many commercial products (Collinge et al., 2022). However, availability is often restricted to individual countries or limited regions (Cordeau et al., 2016), and their efficacy can be inconsistent (Beckerman et al., 2023). Reasonable control of pathogens achieved in controlled research environments has often proved difficult to translate into “on-farm” settings where environmental factors such as humidity and temperature can significantly impact pathogens and biological control agents (BCAs) (Collinge et al., 2022). Commercialization of a BCA can also be challenging. However, despite this, many companies within the agroindustry are now aiming to market new BCA products (Collinge et al., 2022), as the challenge of creating sustainable food production systems with a reduced dependency on agrichemicals is addressed.

In biological control, chemical compounds, particularly secondary or specialized metabolites produced by microorganisms, have gained considerable attention (Glare et al., 2016). Among these compounds, volatile organic compounds (VOCs) stand out for their potential to inhibit plant pathogen growth and activate plant defense mechanisms (Schalchli et al., 2016; Poveda, 2021; Zhao et al., 2022). Notably, 6-pentyl-alpha-pyrone (referred to here as 6-PP), a ketone produced by *Trichoderma* fungi, has emerged as a focal point of interest. This review explores the potential development and utilization of 6-PP as a biopesticide for combatting plant pathogens.

## 2 6-pentyl-alpha-pyrone structure and biosynthesis

**Figure 1:**
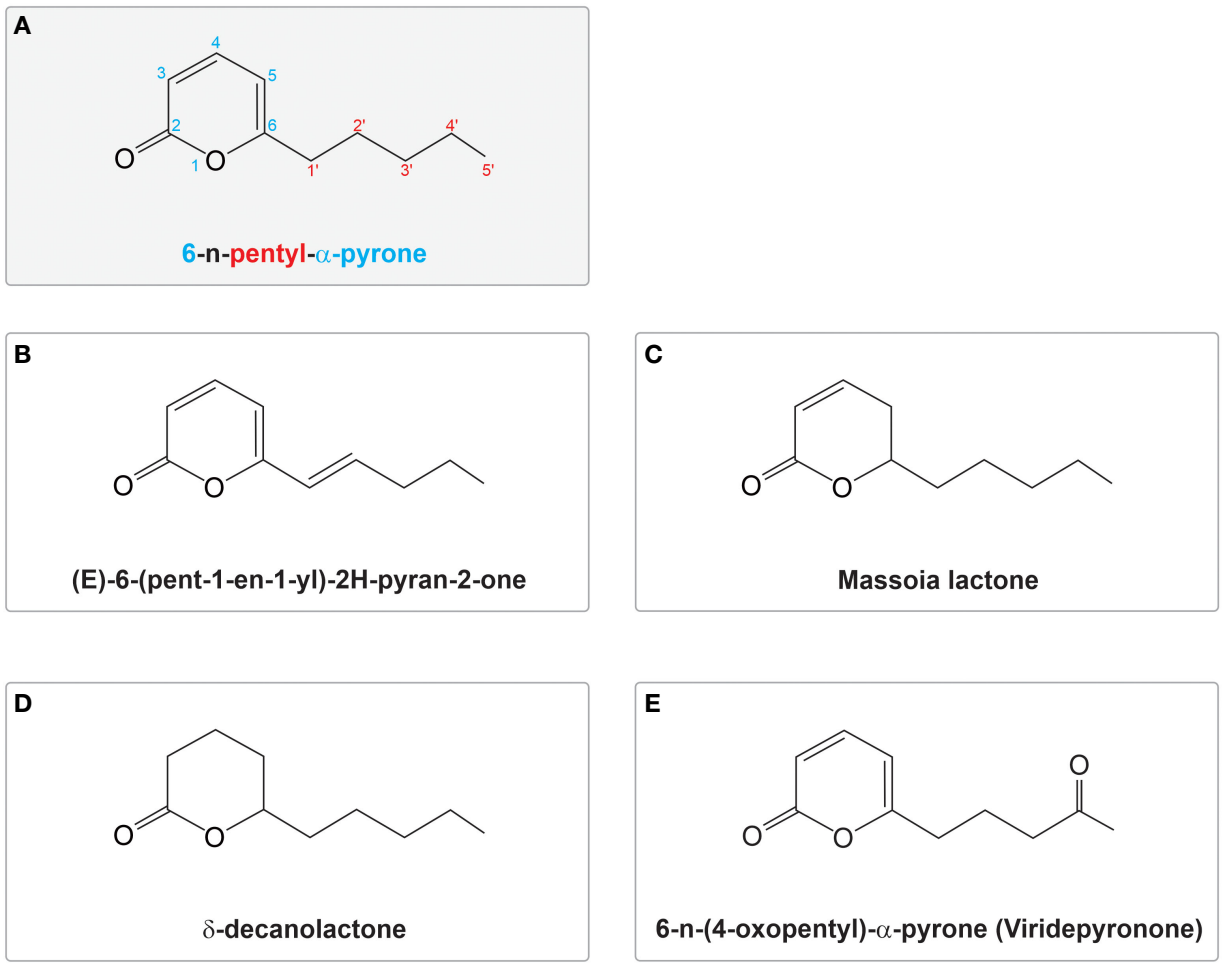
Structure of 6-n-pentyl pyrone (6-PP) and variants reported in *Trichoderma* spp. **(A)** Structural representation of 6-n-pentyl-α-pyrone; the numbers in the molecule represent the position of atoms or functional groups within the molecule. The number ‘6’ indicates the position of the pentyl substituent on the six-membered pyrone ring, shown in blue. The term ‘n-pentyl’ signifies a straight-chain alkyl group containing five carbon atoms, with the numbers in the aliphatic tail highlighted in red. ‘α-pyrone’ describes the structure of the ring, specifying a six-membered heterocyclic ring with a carbonyl group (C=O) at position 2. **(B–E)** Variations of the molecule, including modifications in the ring structure and aliphatic side chain, are shown. These variants illustrate potential structural diversities within the 6PP molecule. The figures were modified from those reported in their original publications (Collins and Halim, 1972; Claydon et al., 1987; Parker et al., 1997; Evidente et al., 2003).

6-PP was first identified by Collins and Halim (1972). It is an organic compound recognized for its organoleptic and flavour characteristics and is one of the early characterized VOCs. This secondary metabolite is an unsaturated, low molecular weight lactone commonly purified from *Trichoderma* cultured media and is produced by various *Trichoderma* species (**Table 1**). Belonging to the pyrone family, 6-PP features a six-membered ring containing an oxygen atom and a carbonyl group. Unlike γ-pyrones, where the carbonyl group is located at the fourth position relative to the oxygen atom within the ring, α-pyrones (also called 2-pyrones) have the carbonyl group in the second position, with 6-PP having a pentyl group attached at the sixth position (Collins and Halim, 1972) (**Figure 1A**). The α-pyrone moiety is widely distributed across various natural sources, including plants, animals, marine life, bacteria, fungi, and insects. It is associated with diverse biological functions, encompassing antifungal, antibiotic, cytotoxic, neurotoxic, and phytotoxic properties (McGlacken and Fairlamb, 2005).

**Table 1:** Species of *Trichoderma* which produce 6-pentyl-α-pyrone.

Species	Reference
*T. viride*	(Collins and Halim, 1972; Moss et al., 1975; Yong et al., 1985; Prapulla et al., 1992; Pezet et al., 1999; Evidente et al., 2003; Abdel-Lateff et al., 2009)
*T. koningii*	(Simon et al., 1988; Worasatit et al., 1994; Cooney and Lauren, 1998; Ismaiel and Ali, 2017).
*T. harzianum*	(Ghisalberti and Rowland, 1993; Scarselletti and Faull, 1994; Cooney et al., 1997a; Cooney and Lauren, 1998)
*T. longibrachiatum*	(Jeleń et al., 2014)
*T. saturnisporum*	(Jeleń et al., 2014)
*T. hamatum*	(Jeleń et al., 2014; Baazeem et al., 2021)
*T. atroviride*	(Serrano-Carreon et al., 1993; Reithner et al., 2005, 2007; Stoppacher et al., 2010; Jeleń et al., 2014; Lee et al., 2016; Cruz-Magalhães et al., 2019; Elsherbiny et al., 2020; Jin et al., 2020; Moreno-Ruiz et al., 2020a; Bello et al., 2022; Cruz-Magalhães et al., 2022; Rao et al., 2022; van Zijll de Jong et al., 2023)
*T. citrinoviride*	(Jeleń et al., 2014)
*T. viridescens*	(Jeleń et al., 2014)
*T. aggressivum*	(Lee et al., 2016).
*T. asperellum*	(Kottb et al., 2015; Lee et al., 2016; Degani et al., 2021; Degani and Gordani, 2022)
*T. gamsii*	(Hong et al., 2014; Lim et al., 2023).
*T. erinaceum*	(Xing et al., 2023).

Four analogues of 6-PP have been isolated from *Trichoderma* species (**Figure 1**) (Evidente et al., 2003; Cruz-Magalhães et al., 2019; 2022; van Zijll de Jong et al., 2023). (E)-6-(pent-1-en1-yl)-2H-pyran-2-one was isolated from various species including *T. harzianum, T. atroviride, T. asperellum*, and *T. viride* (Moss et al., 1975; Claydon et al., 1987; Wickel et al., 2013; Cruz-Magalhães et al., 2019; 2022; van Zijll de Jong et al., 2023). Other 6-PP relatives from *Trichoderma* that have been documented include the saturated variants massoia lactone (Lakhdari et al., 2023) and δ-decanolactone (Reino et al., 2008; Lakhdari et al., 2023), as well as the oxygenated compound 6-(4-oxopentyl)-2H-pyran-2-one, known as viridepyronone in *T. viride* (Evidente et al., 2003) (**Figures 1B–D**). All these variants, including 6-(1g-pentenyl)-2H-pyran-2-one, have exhibited activity against different plant pathogens, including massoia lactone and δ-decanolactone have demonstrated activity against *Penicillium* spp., *Aspergillus fumigatus, Candida albicans*, and *Cryptococcus neoformans* (Claydon et al., 1987; Parker et al., 1997). Viridepyronone, which was identified using spectroscopic methods as 6-(4-oxopentyl)-2H-pyran-2-one showed 90% growth inhibition of *Sclerotium rolfsii* (Evidente et al., 2003). *T. atroviride*, *T. atroviride* “type B”, *T. harzianum* and *T. viride* produce, in addition to 6-PP, minor quantities of another variant identified as (E)-(6-pent-1-enyl)-2H-pyran-2-one (6-pentenyl-pyrone) (Moss et al., 1975; Claydon et al., 1987; Wickel et al., 2013; Cruz-Magalhães et al., 2019, 2022; van Zijll de Jong et al., 2023).

The biosynthetic pathway of 6-PP and other lactones remains to be discovered (Missbach et al., 2023). Initial hypotheses involving linoleic acid metabolism and lipoxygenase enzymes (Serrano-Carreon et al., 1992, 1993) have been challenged (Speckbacher et al., 2020). Alternative proposals suggest the involvement of glutamic acid metabolism or polyketide pathways (Wurz et al., 1988; Sivasithamparam and Ghisalberti, 1998; Lee, 2015). Polyketide and fatty acid biosynthesis share similarities in microorganisms, with polyketide pathways generally recognized as synthesizing most α-pyrones (Wurz et al., 1988; Sivasithamparam and Ghisalberti, 1998).

## 3 6-PP production

### 3.1 Microorganisms producing 6-PP

6-PP’s characteristic coconut aroma has been reported from several *Trichoderma* species (Jeleń et al., 2014), and its chemical structure was first revealed in *T. viride* (Collins and Halim, 1972; Kikuchi et al., 1974), the same as the essence previously identified in peach (Sevenants and Jennings, 1971). Subsequently, it has been isolated and characterized from 13 *Trichoderma* spp. (**Table 1**), and the bacterium *Streptomyces morookaensis* (Zhu et al., 2023). It has been reported to inhibit growth in many plant pathogens (**Table 2**). Studies have shown a strong correlation between *Trichoderma* strains that produce elevated concentrations of 6-PP and their ability to inhibit phytopathogenic fungi (Cruz-Magalhães et al., 2022), thus enhancing crop protection and post-harvest fruit preservation (Worasatit et al., 1994; Elsherbiny et al., 2020). This has promoted research focused on designing screens to identify the best 6-PP producer strains and establish better biocontrol opportunities to protect and improve crop health.

**Table 2:** 6PP activity against different plant pathogens.

Pathogen	6-PP action	References
*Aspergillus flavus, A. niger, A. glaucus*	Strong IMG by DA, medium by V, and weak by D; inhibits mycotoxin production	(Cutler et al., 1986; Claydon et al., 1987)
*Chaetomium cochlioides, C. spinusum*	Strong IMG by D	(Claydon et al., 1987)
*Curvularia lunata*	Weak IMG by D	(Claydon et al., 1987)
*Cylindrocarpon destructans*	Strong IMG by V	(Jin et al., 2020)
*B. cinerea*	Strong IMG by DA and V; Biofumigation protected blueberries; ISG and suppressed infection in kiwifruit	(Claydon et al., 1987; Poole et al., 1998; Bello et al., 2022)
*Bipolaris sorokiniana*	Medium IMG by D	(Simon et al., 1988)
*F. oxysporum, F. culmorum, F. avenaceum, F. cerealis, F. graminearum, F. proliferatum, F. subglutinans, F. acuminatum*	Strong IMG by DA; medium by V and weak by D; ISG with fungistatic activity.	(Claydon et al., 1987; Simon et al., 1988; Scarselletti and Faull, 1994; Ismaiel and Ali, 2017; Rao et al., 2022)
*Gaeumannomyces graminis*	Strong IMG by D, DA, and V	(Claydon et al., 1987; Simon et al., 1988)
*Magnaporthiopsis* *maydis*	Strong IMG by D	(Degani et al., 2021)
*P. litchii*	Strong IMG and ISG	(Xing et al., 2023)
*Phakopsora pachyrhizi*	Strong ISG by D, suppression of Asian soybean rust.	(El-Hasan et al., 2022)
*Phomopsis sclerotioides*	Strong IMG by DA and V	(Claydon et al., 1987)
*Phytophthora cinnamomi*	Strong IMG by D and DA	(Claydon et al., 1987; Simon et al., 1988)
*Phytophthora infestans*	Medium IMG by V	(Elsherbiny et al., 2020)
*Pseudocercosporella herpotrichoides*	Strong IMG by DA and V	(Claydon et al., 1987)
*Pyrenochaeta lycopersici*	Strong IMG by DA and V	(Claydon et al., 1987)
*Pythium ultimum*, *P. middletonii*	Strong IMG by D and DA, and medium by V	(Claydon et al., 1987; Simon et al., 1988)
*Rhizoctonia cereales*	Strong IMG by DA and medium by V	(Claydon et al., 1987)
*Rhizoctonia solani*	Strong IMG by D, protection from lettuce dieback. Reduced sclerotia formation.	(Claydon et al., 1987; Simon et al., 1988; Scarselletti and Faull, 1994; van Zijll de Jong et al., 2023)
*Sclerotium rolfsii*	Suppression of pathogen on lentil seedlings	(Dodd et al., 2000)
*Sclerotinia sclerotiorum*	Strong IMG by DA and weak by V	(Claydon et al., 1987; van Zijll de Jong et al., 2023)
*Verticillium dahlia, V. fungicola*	Strong IMG by DA and V	(Claydon et al., 1987)

Abbreviations used in the table are as follows: IMG, inhibition of mycelial growth; ISG, Inhibition of spore germination; DA, Direct application; D, Diffusion; V, Volatile.

All data published indicate that 6PP has broad spectrum activity against plant pathogenic oomycetes and fungi affecting many economically important crops.

*T. atroviride* and *T. viridescens* are noted for producing higher levels of 6-PP compared to other *Trichoderma* species (Jeleń et al., 2014). However, in a screening of 77 strains encompassing eight *Trichoderma* species, 6-PP was absent in cultures of *T. virens*, *T. harzianum*, and *T. koningii*, with observed intraspecies variations. Notably, 40% of *T. citrinoviride*, 70% of *T. hamatum*, and 40% of *T. viride* strains were found to produce 6-PP. 6-PP production by *T. harzianum* and *T. koningii* may also be strain-specific, as production has been reported by other authors (see **Table 1**). Additionally, it is important to note that isolates of *T. atroviride* were previously classified *as T. harzianum* for an extended period (Jeleń et al., 2014). In nearly all studies of VOCs produced by *Trichoderma* strains, 6-PP was the main VOC produced, reaching concentrations higher than 50% of the total VOCs emitted (Lee et al., 2016; Elsherbiny et al., 2020; Jin et al., 2020; Moreno-Ruiz et al., 2020a; Bello et al., 2022; Cruz-Magalhães et al., 2022; Rao et al., 2022; van Zijll de Jong et al., 2023).

### 3.2 Nutrients regulate the production of 6-PP

*Trichoderma* produces 6-PP when grown in different solid and liquid media, such as minimal medium supplemented with various carbon and nitrogen sources (Moss et al., 1975; Yong et al., 1985; Serrano-Carreon et al., 1993; Bonnarme et al., 1997), malt extract (Claydon et al., 1987), Sabouraud dextrose (Bello et al., 2022), and potato dextrose broth/agar (PDB/PDA) (Collins and Halim, 1972; Simon et al., 1988; Prapulla et al., 1992; Scarselletti and Faull, 1994; Worasatit et al., 1994; Reithner et al., 2005; Lim et al., 2023). In PDB and PDA, *Trichoderma* produces higher 6-PP concentrations (Bello et al., 2022), suggesting that media-rich nutrients promote the primary production of 6-PP. Also, nitrogen sources and cultivation methods impact the production; higher levels were obtained with glutamate, glycine, and ammonium in submerged culture, whereas in surface culture, higher levels were obtained with aspartate and casein (Yong et al., 1985).

**Figure 2:**
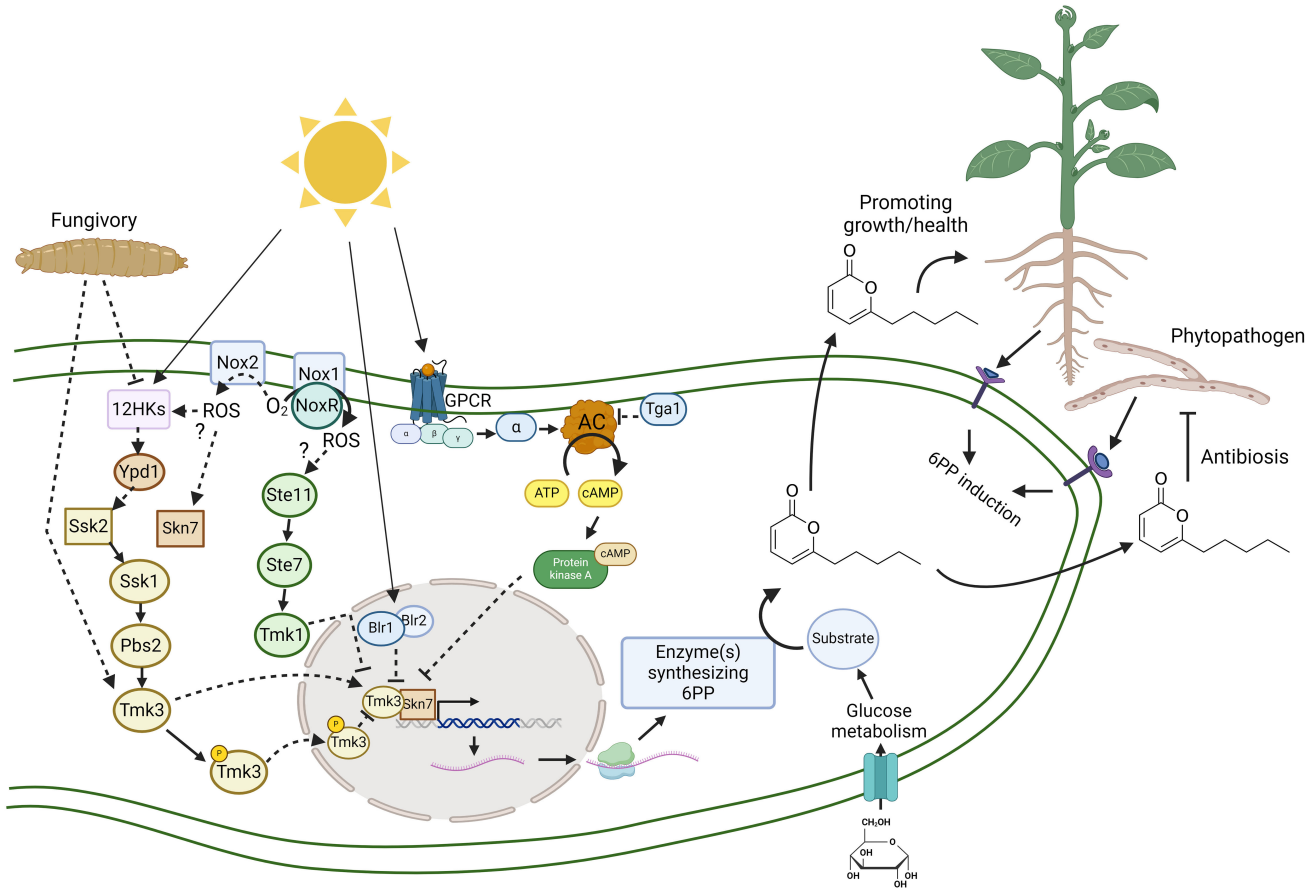
Abiotic and biotic interactions regulating the production of 6-pentyl-α-pyrone in *Trichoderma*. This hypothetical model explains at the molecular level the abiotic (sunlight) and biotic (fungivory, phytopathogens, and plants) interactions involved in regulating the biosynthesis of 6-PP and the main functions in *Trichoderma* interacting with plants or inhibiting phytopathogenic fungi, mechanisms both highly relevant in crop protection. Production of 6-PP is affected in mutant strains lacking in the *tga1* gene encoding Gα protein (Tga1) and thus inhibiting cyclic adenosine monophosphate (cAMP) levels, the Mitogen-Activated Protein Kinase (MAPK) Tmk1 and Tmk3, the response regulator Skn7 and NADPH oxidase 1 (Nox1), 2 (Nox2), and the regulator NoxR. In filamentous fungi, the MAPK Tmk1 is regulated by MAPKK Ste7 and the last is regulated by MAPKKK Ste11, forming a cascade of at least three protein kinases. The cascade MAPKKK Ssk2 – MAPKK Pbs2 – MAPK Tmk3 is regulated by a two-component system consisting of a histidine kinase (HK) sensor, a histidine-containing phosphotransferase (Ypd1), and a response regulator (Ssk2/Skn7) (Schmoll et al., 2016). Blr1 and Blr2 form a blue-light receptor in *Trichoderma* (Schmoll et al., 2010). The heterotrimeric G proteins (α, β, and γ) associated with G protein-coupled receptors (GPCR) regulate cAMP synthesis from ATP by an adenylyl cyclase (AC), a second messenger which regulates protein kinase A (PKA) (Schmoll et al., 2016). Arrows (→) indicate positive regulation, activation, or induction. Bars (⊥) indicate negative regulation, repression, or inactivation.

As a carbon source, glucose promotes higher 6-PP production than glycerol (Bonnarme et al., 1997), suggesting that the metabolism of glucose is involved in providing the precursor molecule to synthesize 6-PP, as was proposed using labelled glucose (Whitaker et al., 1998; **Figure 2**). Also, vegetable oil promotes 6-PP accumulation in the media. However, the mechanism is not understood. It is not clear if fatty acids act as inducers or precursors or promote accumulation as a more compatible hydrophobic solvent as 6-PP is mainly recovered from the oil phase (Bonnarme et al., 1997). Labelled linoleic acid in a *Trichoderma* culture indicated that the metabolism of fatty acids by β-oxidation was the probable pathway to provide the precursor to synthesize 6-PP (Serrano-Carreon et al., 1993). The core metabolism of carbon sources (carbohydrates or fatty acids) appears to provide the precursor/substrate for 6-PP biosynthesis, but this is still not established.

In PDB medium, cultures of *T. viride* (0101 strain) produced a maximum concentration of 110 ppm of 6-PP. Adding this maximum concentration into a fresh medium did not inhibit growth in *T. viride* and did not further increase its 6-PP production, suggesting non-toxicity for the fungus (Prapulla et al., 1992). This observation hints at the possible involvement of a negative feedback loop in regulating 6-PP biosynthesis; a reduction in production of 6-PP after reaching the threshold. However, another *T. viride* strain (TSP2) displayed sensitivity to additional 6-PP in the medium; concentrations surpassing 300 mg/L inhibited mycelial growth (Bonnarme et al., 1997). These findings underscore the existence of variations in both the yield sensitivity and regulation of 6-PP among different strains of *T. viride*. Interestingly, bioactive compounds produced by plant pathogens enhanced 6-PP biosynthesis in *T. atroviride*. In the presence of *Rhizoctonia solani* supernatant, 6-PP production was around 12 times greater than for the control (Flores et al., 2019). This result suggests it may be possible improve 6-PP production by co-culturing with fungal pathogens.

### 3.3 6-PP production during the interaction between *Trichoderma* and phytopathogens

Members of the genus *Trichoderma* are known for parasitizing various phytopathogenic oomycetes and fungi (Worasatit et al., 1994; Elsherbiny et al., 2020; Degani et al., 2021; El-Hasan et al., 2022; Rao et al., 2022; Xing et al., 2023). During such interactions, *Trichoderma* detects the pathogen from a distance and subsequently secretes lytic enzymes, leading to the degradation and eventual demise of the pathogen. Notably, in interactions between *Trichoderma* and phytopathogens such as *B. cinerea* and *Fusarium culmorum*, the production of 6-PP was recorded (Cooney et al., 1997a; Cooney and Lauren, 1998), indicating *Trichoderma’s* ability to detect the presence of pathogen and stimulate 6-PP production (**Figure 2**). This phenomenon suggests a coordinated regulation during parasitic interactions, facilitating a synergistic interplay between metabolites and hydrolytic enzymes. Currently there is no evidence of any negative impacts of 6-PP on beneficial fungi, as FVOCs have yet to be used in large-scale applications (Schulz-Bohm et al., 2017). Furthermore, the impact of 6-PP and other volatile organic compounds on plant pathogens has been investigated mostly in *in vitro* experiments (Jiménez-Bremont et al., 2024), but also in greenhouse studies, and the field (Pascale et al., 2017).

### 3.4 Light and 6-PP production

Environmental cues like light, temperature, and pH can activate secondary metabolite production (Yu et al., 2023). In *T. atroviride*, 6-PP production is negatively regulated by white, blue, green, yellow, or red light but produced in darkness (Moreno-Ruiz et al., 2020a). *Trichoderma* has blue (Blr1/Blr2, Env1 and Cryptochromes), green (opsins), and red and far-red (Phy1) receptors for light. These are proteins associated with chromophores capable of absorbing photons of different wavelengths (Schmoll et al., 2010). Light responses are regulated by the Blue Light Receptor (Blr1/Blr2); the Blr1 protein has a LOV (Light-Oxygen-Voltage) sensor domain, binding a flavin as a chromophore, which associates with Blr2 to form a photoregulatable transcription factor (Schmoll et al., 2010). The Blr1/Blr2 complex and Tmk3 signaling pathway regulate conidia production, cellular stress, metabolism, and growth in the presence of white and blue light (Esquivel-Naranjo et al., 2016; Calcáneo-Hernández et al., 2020) while cryptochromes were not involved in light responses (García-Esquivel et al., 2016) and opsin has yet to be investigated. The MAPK Tmk3 has dual roles regulating 6-PP production in darkness and repression by red light (Moreno-Ruiz et al., 2020a). Interestingly, *Trichoderma* as an endophyte is associated with plant roots in a dark environment, producing 6-PP to protect the plant. In contrast, light could inhibit this chemical communication with plants and promote conidiation for dispersion. In this regard, *T. atroviride* induced 6-PP during interaction with *Arabidopsis thaliana* (Torres-Ortega et al., 2022; **Figure 2**), suggesting that *Trichoderma* senses signals from the plant to establish a chemical dialogue that is more sensitive in darkness, deeply in the rhizosphere, and probably as an endophyte.

In *T. atroviride*, a Gα protein Tga1 negatively regulates cAMP production; a mutant lacking in *tga1* accumulated high cAMP levels, but production of 6-PP was reduced (Reithner et al., 2005). Adenylyl cyclase synthesizes cAMP using adenosine triphosphate (ATP), and its activity is regulated by G protein-coupled receptors (GPCR) and heterotrimeric G proteins (**Figure 2**). Light and nutrients regulate cAMP level, which activates the protein kinase A (Casas-Flores et al., 2006). Considering that light stimulates cAMP accumulation (Casas-Flores et al., 2006), this might be another molecular mechanism that could affect the production of 6-PP (Reithner et al., 2005). This evidence suggests that light could regulate the biosynthesis of 6-PP through many different signaling pathways in *Trichoderma* (**Figure 2**), restricting 6-PP production in darkness.

### 3.5 Mitogen-active protein kinases and 6-PP

*Trichoderma* has three MAPK (Tmk1, Tmk2 and Tmk3) pathways to interact with the environment. The MAPK Tmk3 has a crucial role in integrating stress, light and mycoparasitism (Esquivel-Naranjo et al., 2016; Moreno-Ruiz et al., 2020a). A mutant lacking in Tmk3 has reduced 6-PP production in darkness, but red light did not repress its production (Moreno-Ruiz et al., 2020a; **Figure 2**), suggesting a dual role as an activator in darkness and a repressor in light. Also, in *T. atroviride*, Tmk3 induced 6-PP during fungivory as a defense mechanism against *Drosophila melanogaster* larvae making *Trichoderma* less attractive to be depredated (Atriztán-Hernández et al., 2019). It would be interesting to explore whether 6-PP and other VOCs act as repellents, deterring pests from crops or making them less attractive targets.

*Trichoderma* has 12 histidine kinase (HK) sensors which might sense diverse cues, including light and predators to regulate the cascade of the MAPKKK Ssk2- MAPKK Pbs2- MAPK Tmk3 pathway by transient phosphorylation (**Figure 2**), and consequently cellular conidiation, stress and 6-PP production (Esquivel-Naranjo et al., 2016; Cruz-Magalhães et al., 2022; Calcáneo-Hernández et al., 2023). In fungi, red and far-red light are sensed by a phytochrome Phy1 (Schmoll et al., 2010), a HK sensor, which regulates orthologous to the MAPK Tmk3 (Yu et al., 2016). The response regulator Skn7 regulates 6-PP production; however, mutants lacking the response regulator Ssk1 produced higher 6-PP concentrations (Cruz-Magalhães et al., 2022). MAPK Tmk3 is concentrated in the nucleus under cellular stress or light (Esquivel-Naranjo et al., 2016), and Skn7 is a transcription factor with a nuclear signal (Cruz-Magalhães et al., 2022), indicating that both proteins could regulate expression of genes encoding enzymes involved in synthesis of 6-PP (**Figure 2**). Although phenotypes of mutants lacking in Tmk3 and Skn7 differ in stress tolerance and conidia production, they could be working together to regulate 6-PP production, antagonism, and probably interaction with the plant.

MAPK Tmk1 negatively regulated 6-PP production as a mutant lacking in Tmk1 produced higher levels of 6-PP (Reithner et al., 2007). Also, Tmk1 is involved in mycoparasitism. However, 6-PP levels during the *Trichoderma*-host interaction were not analyzed, and players upstream of the cascade of the MAPKKK Ste11- the MAPKK Ste7 – the MAPK Tmk1 are still unknown (**Figure 2**).

### 3.6 Reactive oxygen species and 6-PP

Reactive oxygen species (ROS) are highly reactive molecules derived from oxygen, capable of damaging cellular components including proteins, lipids, carbohydrates, nucleic acids, and several other small organic molecules (Cruz-Magalhães et al., 2019). ROS are essential as secondary messengers in various cellular physiological and biochemical processes. The overexpression of PR genes, such as chitinases, glucanases, thaumatin, and defensin proteins, either individually or in combination, significantly enhances the plant’s defense response against a broad spectrum of pathogens (Ali et al., 2018). *Trichoderma* has two NADP oxidases (Nox1 and Nox2), which produce ROS under the regulation of NoxR (Hernández-Oñate et al., 2012; Cruz-Magalhães et al., 2019). Nox2 has not been demonstrated to produce ROS, but the *nox2* mutant reduced 6-PP production and displayed less antagonism to *Rhizoctonia solani* and *Sclerotinia sclerotiorum* when exposed to volatiles from a distance. The opposite was demonstrated in the *nox*1 mutant, which produced higher 6-PP levels and was a better antagonist against the same phytopathogens (Cruz-Magalhães et al., 2019). These pieces of evidence suggest that Nox2 is an activator while Nox1 has a negative role in 6-PP production and antagonism in *Trichoderma*. ROS produced by Nox1/NoxR regulates conidiation induced by mechanical injury and phosphorylation of Tmk3 (Hernández-Oñate et al., 2012). Tmk1 and Tmk3 regulate mechanical injury responses in *T. atroviride* (Medina-Castellanos et al., 2014). Whether an interplay between Tmk1 and Nox1 negatively regulates 6-PP production and Tmk3, Skn7 and Nox2 necessary for 6-PP production requires further investigation (**Figure 2**). Surprisingly, temperature effects on the output of 6-PP have not been reported. Most studies have used a range between 25°C to 28°C (Sarhy-Bagnon et al., 2000; Ramos et al., 2008).

## 4 Effect of 6-PP on plant pathogens

6-PP has potent antifungal activities, disrupting fungal growth and pathogen development (Degani et al., 2021; Elhamouly et al., 2022; Hao et al., 2023). In a dose-response experiment, Hao et al. (2023) showed that 25 mg/L of 6-PP in Hoagland nutrient solution inhibited *F. oxysporum* growth by up to 70%. In comparison, at 10–20 mg/L, 6-PP had no effect on growth but reduced fusaric acid production by the fungal pathogen. The compound might interfere with key physiological processes in fungi, including cell wall synthesis and membrane integrity, which can inhibit growth (Reithner et al., 2007; Moreno-Ruiz et al., 2020b, 2021). Kottb et al. (2015) showed that 6-PP acts as a signal molecule to inhibit the growth of *Botrytis cinerea* and *Alternaria brassicicola* in *Arabidopsis thaliana*. Another study showed that 6-PP induces systemic resistance in *Nicotiana tabacum* plants against *Tobacco mosaic virus* (Taha et al., 2021).

**Figure 3:**
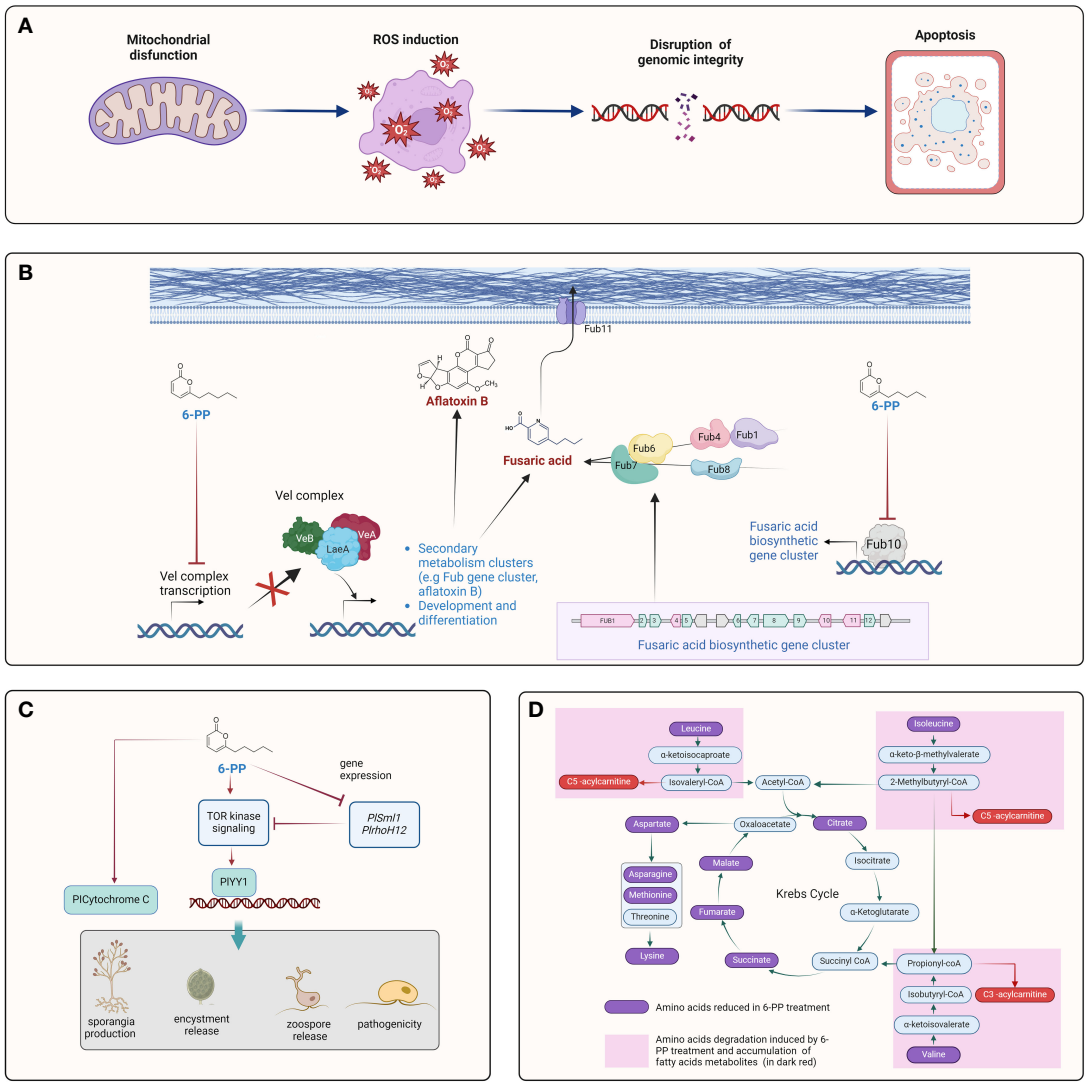
Cellular processes targeted by 6-PP on plant pathogens. The figure illustrates various aspects of the interaction between 6-PP and plant pathogens. **(A)** A mitochondrial dysfunction may provoke ROS imbalance and affects genome integrity, leading to the programed cell dead (Apoptosis) (Liu et al., 2023; Xing et al., 2023). **(B)** 6-PP regulates production of mycotoxin in plant pathogens by downregulation of *vel* complex and *fub10* genes, key players to regulate expression of the cluster Fub and synthesis of aflatoxin. (Niehaus et al., 2014; Ismaiel and Ali, 2017; Hao et al., 2023) **(C)** 6-PP regulates the TOR signaling pathway, impacting different development process such as growth, sporangium formation, encystment, zoospore release and pathogenicity (Wu et al., 2023). **(D)** Alteration of organic acids metabolism via the Krebs cycle may affect the physiology of plant pathogens. 6-PP alters metabolic pathways in plant pathogens, affecting their ability to acquire nutrients and energy for growth and survival (Jin et al., 2020).

6-PP has demonstrated significant efficacy against various plant pathogenic organisms, including fungi, bacteria, viruses, and nematodes (**Table 2**), exerting its effects through direct and indirect mechanisms (**Figure 3A**). Its broad spectrum of activity is noteworthy, encompassing the suppression of economically significant plant pathogens and the activation of plant defense mechanisms upon application to affected tissues. In the subsequent sections, we will delve into the diverse roles of this molecule in combating various pathogens and shed light on its mechanisms of action. Furthermore, we will explore how 6-PP impacts plant modification and its role in inducing systemic resistance against plant pathogens in systemic tissues.

Various studies have demonstrated the efficacy of 6-PP against a diverse array of fungal species. Specifically, it has shown inhibitory effects on *F. moniliforme* (El-Hasan et al., 2007), *Rosellinia necatrix* (Arjona-Girona et al., 2014), *Athelia rolfsii* (Dodd et al., 2000), multiple strains of *Aspergillus flavus*, *Aspergillus parasiticus, Penicillium expansum, F. acuminatum* (Ismaiel and Ali, 2017), and *R. solani* (Claydon et al., 1987; Worasatit et al., 1994). Furthermore, 6-PP has been found to inhibit the filamentous growth of *F. oxysporum f.* sp. *lycopersici*, and *Magnaporthiopsis maydis* and spore germination of *F. oxysporum* (Scarselletti and Faull, 1994; Degani et al., 2021; Degani and Gordani, 2022). It has also reduced hyphal growth of the pathogens *Macrophomina phaseolina, R. solani*, *Sclerotium rolfsii*, *F. oxysporum, F. verticillioides* and *F. moniliforme* (El-Hasan et al., 2007; Ahluwalia et al., 2014, 2015), *B. cinerea* (Poole et al., 1998) as well as two pathogens affecting grapevine trunks, *Eutypa lata* and *Neofusicoccum parvum* (Mutawila et al., 2016).

Claydon et al. (1987) examined the susceptibility of several plant pathogens to 6-PP (**Table 2**). While their analysis revealed that most were susceptible to 6-PP, there were certain exceptions. For example, *Pythium ultimum*, *S. sclerotiorum*, and *R. cerealis* could partially resist the compound. Additionally, disparities in susceptibility were observed among different anastomosis groups of *R. solani*, with strains from AG1 and AG2 displaying higher susceptibility than those from AG4 (Claydon et al., 1987).

The growth of mycelium and the germination of ascospores/conidia in *E. lata*, *N. austral, N. parvum*, and *Phaeomoniella chlamydospora* were suppressed by 6-PP, with the inhibition being correlated with its concentration (Mutawila et al., 2016). Mutawila et al. (2016) found that 6-PP was more effective at inhibiting the ascospores/conidia germination in grapevine trunk pathogens than inhibiting mycelium growth. However, its effectiveness in inhibiting mycelial growth varied across fungal pathogens (Mutawila et al., 2016). As pruning wound infections primarily start with conidia or ascospores, the strong suppression of their germination by 6-PP suggests potential protection for *Trichoderma*-treated wounds. Nonetheless, the limited impact on mycelial growth implies that 6-PP-producing *Trichoderma* strains may not fully eradicate established pathogens in pruning wounds (Mutawila et al., 2016). Improving wound protection might involve extending the interval between applying Trichoderma biocontrol agents and pathogen inoculation (Munkvold and Marois, 1993; John et al., 2008).

Oomycetes, a highly destructive group of plant pathogens, can transform fields of thriving crops into desolate landscapes of blackened stalks within days, profoundly impacting agriculture. *Phytophthora infestans*, notorious for triggering the Irish famine through potato late blight, is among the most destructive (McGowan and Fitzpatrick, 2020). Additionally, *P. cinnamomi* poses a global menace, infecting nearly 5000 plant species across agriculture, forestry, and horticulture, and can cause widespread devastation (Jung et al., 2013; Hardham and Blackman, 2018).

Interestingly, while two VOCs produced by *T. atroviride*, namely 3-methyl-1-butanol (isoamyl alcohol) and 2-methyl-1-propanol (isobutyl alcohol), inhibited the growth of *P. infestans*, 6-PP had a lower activity with only 34% inhibition compared to the 100% inhibition achieved by the other two VOCs (Elsherbiny et al., 2020). This suggests the possibility of differential roles of specific VOCs, such as 6-PP, in the anti-oomycete activity of *T. atroviride*. Claydon et al. (1987), reported that 6-PP isolated from *T. harzianum* exhibited inhibitory properties against *P. cinnamomi*. Contrary to its low activity against *P. infestans*, the oomycete *Peronophythora litchii* is highly susceptible to 6-PP (Xing et al., 2023). 6-PP caused significant inhibition of the oomycete’s growth and sporangial germination, resulting in severe cellular and intracellular destruction. This suggests a contrasting effect of 6-PP on different oomycete species. Recent studies have also suggested the involvement of specific signaling transduction pathways in the interaction of 6-PP with fungi and oomycetes (Xing et al., 2023). For instance, in *P. litchii*, 6-PP triggers the upregulation of genes associated with the Ser/Thr protein kinase target of the rapamycin (TOR) pathway, such as PlCytochrome C and the YinYang1 transcription factor, while concurrently downregulating potential negative regulators of this pathway like PlSpm1 and PlrhoH12 (Wu et al., 2023). These findings imply that 6-PP might inhibit the vegetative growth, sporulation, cyst formation, and pathogenesis of *P. litchii* by modulating the activation of the TOR pathway (**Figure 3C**). This suggests that 6-PP could play a significant role in influencing critical regulatory pathways within these organisms.

While 6-PP showed no antibacterial activity against several bacterial strains, it exhibited positive inhibitory activity against *Staphylococcus aureus*, at a concentration of 100 μg/mL (Ismaiel and Ali, 2017). Upon treatment with 6-PP, bacterial cells displayed notable changes, including diluted cytoplasm and separation of the cell membrane from the cell wall, creating a broad space in these two structures and the appearance of ghost cells lacking a surrounding cell wall (Ismaiel and Ali, 2017).

Biofilms act as a defensive barrier for bacteria, shielding them from the host’s immune responses and unfavorable environmental factors. Interestingly, 6-PP exhibits antibiofilm activity, aiding in the infection of X1ccg1, a lytic bacteriophage of *Xanthomonas campestris* pv. *campestris* (Papaianni et al., 2020). Gram-negative bacteria, particularly those belonging to the *Ralstonia* and *Xanthomonas* genera, are widely recognized as highly destructive plant pathogens with significant economic consequences globally (Scherf et al., 2010; Timilsina et al., 2020). For instance, *R. solanacearum* and *X. campestris* cause substantial damage to crops like tomatoes and peppers, resulting in yield losses of up to 50% in numerous countries worldwide.

In an *in vitro* study, Yang et al. (2012) showed that 6-PP exhibited potent nematicide activity, causing mortality rates exceeding 90% in *Panagrellus redivivus* and *Caenorhabditis elegans* within 48 hours at a concentration of 200 mg/L. Similarly, it resulted in 87% mortality of *Bursaphelenchus xylophilus* within the same timeframe and at the same concentration.

### 4.1 Mode of action of 6PP on plant pathogens

#### 4.1.1 Disruption of cell membranes and modification of cell structure in plant pathogens

6-PP disrupts fungal and oomycete cell membranes by interacting with the lipid bilayer structure. The compound’s lipophilic nature allows it to penetrate the membrane’s hydrophobic interior. Once inside, 6-PP disturbs the arrangement of lipids and proteins, compromising the integrity of the membrane (Ismaiel and Ali, 2017). The most frequent alteration in fungi observed was related to the detachment of the plasma membrane from the cell wall (Ismaiel and Ali, 2017). This disruption may increase permeability, allowing ions, metabolites, and other cellular components to leak out of the cell. As a result, the pathogen may experience osmotic imbalance, cellular dehydration, and eventual lysis. Moreover, 6-PP may specifically target membrane-associated proteins or enzymes, further disrupting membrane function. For example, it may interfere with ion channels or transporters essential for maintaining cellular homeostasis (Cornelius et al., 2015). The disruption of membrane integrity impairs the pathogen’s structural integrity. It hinders its ability to interact with the external environment and acquire nutrients, ultimately leading to growth inhibition and cell death.

In *P. litchii*, the agent causing litchi downy blight, 6-PP triggered widespread disruption in organelles, including mitochondrial breakdown and vacuole swelling, leading to their collapse. Higher 6-PP concentrations exacerbated cellular damage, causing increased vacuolation, thickened cell walls, and membrane disintegration. Additionally, 6-PP significantly hindered sporulation, sporangium germination, and mycelial growth, resulting in desiccation and distortion of mycelia, sporangiophores, and irregular sporangia surfaces. Ultimately, mycelia and sporangiophores collapsed entirely, with sporangia severely shrinking (Xing et al., 2023).

*Clarireedia* spp. is a fungal pathogen causing dollar spot in turf-bent grass, ultimately leading to extensive patchiness. 6-PP effectively inhibited *C. jacksonii*, triggering significant morphological alterations and oxidative damage. Furthermore, it disrupted energy metabolism within the fungus and promoted cellular apoptosis (Liu et al., 2023). A transcriptome analysis of *C. jacksonii*, treated with 6-PP, revealed the accumulation of transcripts related to lipid metabolism and malondialdehyde (MDA) synthesis, suggesting a response to oxidative and oxidoreduction stress induction.

#### 4.1.2 Inhibition of enzymes

Based on findings in other organisms, such as mammal systems, 6-PP may exert inhibitory effects on specific enzymes critical for plant pathogens’ metabolic processes and survival. In mammal systems, 6-PP is considered a “phospholipid antagonist”, which inhibits the activity of alpha-Na+, K+ATPase, which plays an essential role in muscle contraction and inotropic effects of cardiac glycosides (Kapri-Pardes et al., 2011). In fungi, the proteins equivalent to Na+, K+ATPases are the proton-ATPases involved in regulation of hypersensitivity to osmotic stresses, cell wall stressors and oxidative stress (Mou et al., 2020; Deng et al., 2023). In fungi, 6-PP may also target other enzymes involved in critical metabolic pathways, such as glycolysis, the tricarboxylic acid (TCA) cycle, and fatty acid biosynthesis as well as key components associated with nutrients sensing such as TOR kinase signaling (Jin et al., 2020; Lim et al., 2023; Liu et al., 2023; Wu et al., 2023). By inhibiting enzymes within these pathways, 6-PP disrupts the production of essential metabolites and intermediates, impairing the pathogen’s ability to generate energy and synthesize macromolecules necessary for growth and replication (Jin et al., 2020). Although 6-PP can be toxic to plant pathogens and pests, it has been reported to be safe to non-target organisms, including birds, wild mammals, aquatic organisms, non‐target soil arthropods, earthworms, and other soil macro‐ and microorganisms (Alvarez et al., 2022).

#### 4.1.3 Induction of oxidative stress

Exposure to 6-PP induces oxidative stress within plant pathogens by promoting the generation of ROS such as superoxide radicals (O_2_•-) and hydrogen peroxide (H_2_O_2_) within the cells. The production of ROS overwhelms the pathogen’s antioxidant defense mechanisms, leading to oxidative damage and cellular dysfunction. ROS-mediated damage disrupts essential cellular processes, such as enzyme function, DNA replication, and signal transduction, ultimately leading to growth inhibition and cell death. Additionally, prolonged exposure to oxidative stress may trigger apoptotic-like programmed cell death pathways in the pathogens, further exacerbating their demise (Redza-Dutordoir and Averill-Bates, 2016).

In the genus *Clarireedia*, exposure to 6-PP induces mitochondrial dysfunction, resulting in damage to cell membranes and mitochondria, which leads to excessive ROS production and subsequent oxidative stress, ultimately inhibiting fungal proliferation. This rise in ROS triggers an increase in alcohol dehydrogenase (ADH) and decrease in aldehyde dehydrogenase (ALDH), causing aldehyde accumulation. Aldehydes disrupt cell homeostasis, deactivate enzymes, and cause DNA damage and cell death in *C. jacksonii* (Liu et al., 2023). Additionally, 6-PP upregulates detoxification-related genes, particularly Superoxide dismutase (SOD), catalase (CAT), and genes related to glutathione metabolism and Glutathione S-transferase (GST), indicating an oxidative stress response. By using two strains of *C. jacksonii* with varying sensitivity to 6-PP, it was observed that resistant strains (101) exhibited higher SOD and CAT activities compared to sensitive strains, suggesting specific differences in response to 6-PP-induced toxicity (Liu et al., 2023).

#### 4.1.4 Alteration of gene expression

6-PP has been shown to modulate gene expression in plant pathogens, leading to alterations in their physiological responses and virulence attributes. The compound may influence the transcriptional regulation of genes involved in stress responses, cell wall integrity, toxin production, and other virulence factors. By affecting gene expression, 6-PP can disrupt the coordination of cellular processes essential for survival and pathogenicity (Mutawila et al., 2016; Jin et al., 2020; Hao et al., 2023).

Soil-less cultivation methods like hydroponics and substrate culture offer an alternative to traditional soil-based plant growth. When 6-PP was incorporated into soil-less solutions, it effectively suppressed Fusarium wilt of tomato caused by *Fusarium* spp., significantly reducing the disease index compared to the untreated control (Hao et al., 2023). Additionally, 6-PP induced the downregulation of toxin biosynthesis and transport encoding genes (such as FUB1, FUB4, and FUB10), as well as growth-related genes (VelA, VelB, and LaeA), resulting in decreased growth and hyphae formation of *F. oxysporum* (Hao et al., 2023) (**Figure 3B**).

The production of 6-PP in *T. atroviride* depends on the specific strain employed (van Zijll de Jong et al., 2023). For instance, when *T. atroviride* strains UST1 and UST2 were cultivated alongside the grapevine trunk pathogens *E. lata* and *N. parvum*, different outcomes emerged in a minimal defined medium and a grapevine cane-based medium (GCBM). Co-cultivation of UST1 with *N. parvum* resulted in increased 6-PP production in both media. Conversely, the presence of the two trunk pathogens either reduced or minimally impacted 6-PP production by UST2, which predominantly occurred in the GCBM (Mutawila et al., 2016).

6-PP holds promise as a potent fungicidal agent against *Cylindrocarpon destructans* (Jin et al., 2020). The exposure to 6-PP led to notable alterations in eight key pathways within *C. destructans*, including amino acid metabolism, glutathione metabolism, and butanoate metabolism. These pathways regulate and produce metabolites, highlighting the broad impact of 6-PP stress on fungal metabolism (Jin et al., 2020). The observed reduction in the accumulation of metabolites associated with the citric acid cycle or Krebs cycle, including malic, succinic, citric, and fumaric acid, in the presence of 6-PP implies inhibition of these cycles by 6-PP (**Figure 3D**) (Jin et al., 2020).

ECHS1, an enoyl-CoA hydratase, is significantly suppressed at the transcriptional level in response to 6-PP exposure (Jin et al., 2020). This gene was identified as central in 6-PP-induced stress through a constructed gene co-expression network. Inhibition of enoyl-CoA hydratase in fungi can disrupt fatty acid metabolism, leading to energy deprivation, compromised cell membrane integrity, impaired growth, and heightened susceptibility to environmental stresses. Furthermore, autophagy was observed in *C. destructans* cells under 6-PP stress conditions. In summary, 6-PP triggers autophagy in *C. destructans* by transcriptionally downregulating ECHS1 expression and inhibiting ECHS1 protein function (Jin et al., 2020).

In *Clarireedia jacksonii* treated with 6-PP there was an upregulation of the fatty acid degradation pathway which contributed to fungal cell apoptosis initiation. Transcriptome analysis highlighted disturbances in cell wall and membrane integrity, disruptions in energy metabolism, and an overabundance of ATP production, leading to fungal apoptosis. These findings provide valuable insights into potential disease management strategies (Liu et al., 2023).

#### 4.1.5 Inhibition of biosynthetic pathways of plant pathogens

VelA, VelB, and LaeA are pivotal growth-related genes that orchestrate fungal development, secondary metabolism, and adaptation to environmental cues. Their intricate regulatory networks influence various aspects of fungal biology, making them important targets for understanding and manipulating fungal growth and behavior. In *F. oxysporum* HF-26, 6-PP caused downregulation of VelA, VelB, and LaeA genes, reducing *F. oxysporum* mycelial growth and hyphae formation (**Figure 3B**) (Hao et al., 2023). In *F. fujikuroi*, the fungal-specific velvet complex Vel1 (VelA) and Lae1 (LaeA) regulates fusaric acid production (Niehaus et al., 2014). 6-PP derived from *T. harzianum* has been shown to suppress fusaric acid production from *F. moniliforme* (El-Hasan et al., 2008). Furthermore, treatment of *F. oxysporum* HF-26 with 6-PP resulted in the downregulation of crucial genes within the fusaric acid gene cluster, including FUB1 (Polyketide synthase), FUB4 (serine hydrolase), and FUB10 (Fungal-type Zn(II)_2_Cys_6_ transcription factor), which are associated with toxin biosynthesis, along with FUB11, encoding the transport gene (Hao et al., 2023). FUB10 serves as a vital positive regulator of the FUB gene cluster (Brown et al., 2015; Studt et al., 2016). This may suggest that 6-PP could regulate the VelA/VelB/LaeA complex, and the gene regulated by the complex (**Figure 3B**). Furthermore, 6-PP exhibited inhibitory effects on aflatoxin B1 (AFB1) production in various strains of *A. flavus* and *A. parasiticus* cultured in a liquid medium (**Figure 3B**) (Ismaiel and Ali, 2017) (**Figure 3B**). The findings demonstrated the significant efficacy of 6-PP in suppressing AFB1 production, resulting in reductions ranging from 34% to 55%.

## 5 The role of 6-PP in plants

### 5.1 Molecular mechanisms

Previous studies have linked the induction of plant defense responses to 6-PP derived from *Trichoderma* spp (El-Hasan et al., 2007). Despite the considerable body of literature exploring the effects of 6-PP on plants, such as changes in gene expression, enzymes activities, and metabolic pathways, there remains a scarcity of information regarding the molecular mechanisms through which these secondary metabolites exert their bioactivity.

To address this knowledge gap, further investigation is warranted to unravel the intricate signaling pathways and molecular interactions involved in the bioactivity of 6-PP in plants. Techniques such as transcriptomics, proteomics, and metabolomics offer promising avenues for identifying essential genes, proteins, and metabolites influenced by 6-PP. Additionally, exploring specific signaling molecules, receptors, and downstream targets associated with 6-PP-induced responses can provide valuable insights into its mode of action.

Understanding the molecular mechanisms governing 6-PP’s bioactivity in plants is crucial for advancing scientific knowledge and can potentially drive the development of innovative strategies for crop enhancement and sustainable agriculture. By deciphering the molecular pathways through which 6-PP influences plant growth, development, and stress responses, researchers can leverage its beneficial effects to improve crop productivity and resilience in environmental challenges.

6-PP might not only act as a defense response signal but also as a messenger, which activates the defense responses in crops (Carillo et al., 2020; Esparza-Reynoso et al., 2020). The involvement of plant defenses is the main reason why plants can better protect themselves and combat pathogen attacks. The complex relationship between the compound and microbial groups in the soil is another key element since soil microbiome modifications can alter the plant pathogen population. For instance, besides protecting banana plants from *F. oxysporum* f. sp. c*ubense*, 6-PP has been found to stimulate beneficial rhizosphere microbiomes to promote banana growth (Zhu et al., 2023). These beneficial microbiomes may also be able to reduce the impacts of some plant pathogens.

### 5.2 Auxin-like activity

In plants, including wheat, maize, tomato, and *Arabidopsis thaliana*, 6-PP has an inhibitory effect at high concentrations and a growth promotion effect at low concentrations (El-Hasan and Buchenauer, 2009; Garnica-Vergara et al., 2016; Mazzei et al., 2016). Sprayed tomato plants with low concentrations of 6-PP produced vigorous growth and an extensive root system (Vinale et al., 2008). A similar situation was observed when 6-PP was applied directly with half-strength Murashige and Skoog solution medium (Mazzei et al., 2016).

When applied externally at low concentrations, such as 1 ppm for root applications or 1 µM for foliar applications, 6-PP demonstrated an auxin-like effect in various plant species, including wheat and tomato seedlings, etiolated pea stems, and substrates containing canola or tomato seeds. This resulted in the enhancement of plant growth and development (Vinale et al., 2008). In lupins immersed in 6-PP there was a significant increase in the plantlets’ biomass when applied at 1 mg/L compared to the control, but no increase in the biomass at 10 mg/L (Arjona-Girona et al., 2014).

Immersion of etiolated pea stems in 1µM of 6-PP induced a positive response (split curvature) compared to the controls (water- and indoleacetic acid-treated stems). Moreover, foliar spray with 1 µM of 6-PP (0.166 mg/L) significantly increased plant height and leaf area, with plants being more developed and vigorous than the controls (Vinale et al., 2008).

6-PP enhanced plant growth and regulated root structure in experiments conducted with *Arabidopsis thaliana* as the experimental model. 6-PP inhibits primary root growth while encouraging lateral root development (Garnica-Vergara et al., 2016). Additionally, 6-PP was seen to impact the expression of PIN auxin-transport proteins in primary roots in a manner dependent on its concentration. The response of lateral roots to 6-PP was found to be influenced by various auxin receptors including TIR1, AFB2, and AFB3, as well as transcription factors ARF7 and ARF19. Furthermore, the perception of 6-PP in primary roots was shown to be modulated by EIN2 (Garnica-Vergara et al., 2016).

### 5.3 6-PP effects on the metabolome

**Figure 4:**
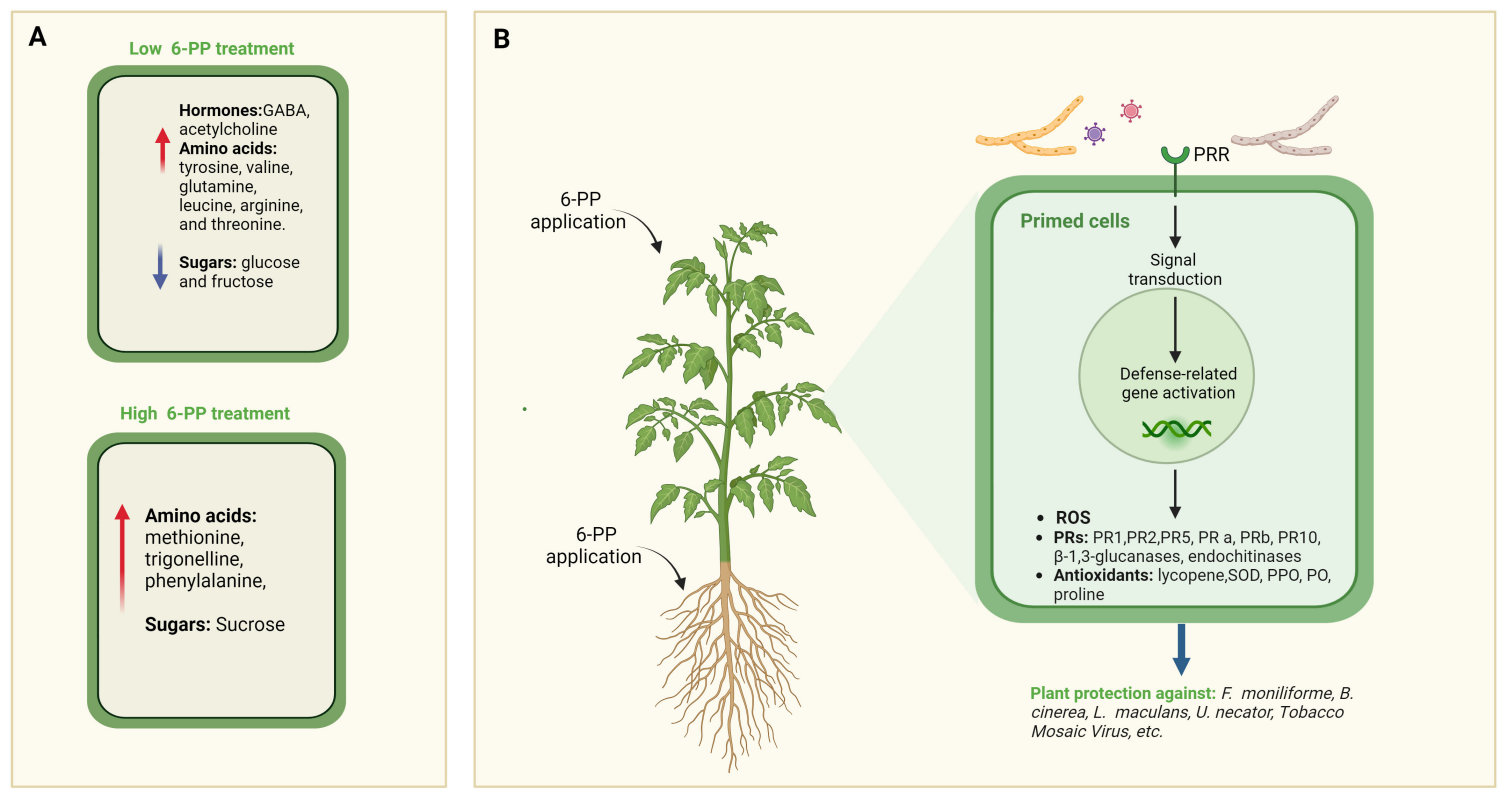
Effects of 6-PP Application on Systemic Tissues. **(A)** Impact of two concentrations of 6-PP on metabolite accumulation in tissues of treated plants. Through metabolomic analysis, distinct accumulation patterns of various metabolites, including secondary metabolites and signalling molecules, are observed in response to the different concentrations of 6-PP. This differential accumulation suggests a dosage-dependent effect of 6-PP on plant metabolism, highlighting its potential role in modulating biochemical pathways involved in stress responses and defence mechanisms (Mazzei et al., 2016). **(B)** depicts the induction of salicylic acids (SA) and associated metabolites, including pathogenesis-related (PR) proteins, in different plant species following treatment with 6-PP. PRR, Pathogen Recognition Receptor.

Intriguingly, the treatment of tomato plants in an axenic medium induced alterations in the metabolome of their leaves. Specifically, exposure to a minimal concentration (0.1µM) of 6-PP resulted in a notable increase in the levels of Gamma-aminobutyric acid (GABA), acetylcholine, and several amino acids, including tyrosine, valine, glutamine, leucine, arginine, and threonine, while glucose and fructose levels decreased. Conversely, exposure to higher concentrations (10μM) led to elevated levels of methionine, trigonelline, phenylalanine, and sucrose (Mazzei et al., 2016). Of particular interest is trigonelline, a plant hormone with diverse regulatory functions, including roles in plant cell cycle regulation, nodulation (Phillips et al., 1992), and mitigation of oxidative stress, thereby supporting plant survival and growth (**Figure 4A**).

### 5.4 6-PP-induced systemic resistance

Induced resistance can be categorized into systemic acquired resistance (SAR) and induced systemic resistance (ISR), each distinguished by the nature of the elicitor and the regulatory pathways involved. SAR typically emerges systemically following localized exposure to pathogen infection or treatment with synthetic or natural compounds. It relies on salicylic acid accumulation, which triggers the expression of numerous pathogenesis-related (PR) genes through the redox-regulated protein NPR1 (non-expression of pathogenesis-related gene 1). In contrast, ISR is commonly induced by root colonization by beneficial bacteria and operates through a pathway sensitive to jasmonate or ethylene. Although ISR activation does not coincide with PR gene expression, it still necessitates NPR1 activity (Pieterse et al., 1998). NPR1’s involvement in salicylic acid signaling is associated with a nuclear function, whereas in jasmonate/ethylene signaling, it pertains to a cytosolic function (Taha et al., 2021).

Salicylic acid is a key signaling molecule in plant defense responses against pathogens in shoots and is required for the microbial community establishment in the roots (Lebeis et al., 2015). The upregulation of SA and PR proteins indicates the activation of systemic acquired resistance (SAR) in plants treated with 6-PP, which enhances their ability to defend against pathogen attacks. Furthermore, the correlation between the accumulation of these metabolites and the observed inhibition of specific pathogens provides compelling evidence of the effectiveness of 6-PP in priming plant defenses and conferring resistance to disease (Vinale et al., 2008; El-Hasan and Buchenauer, 2009; Arjona-Girona et al., 2014; Pascale et al., 2017; Ali et al., 2018; Carillo et al., 2020; Taha et al., 2021; Hao et al., 2023).

The application of 6-PP to seeds or seedlings by immersion in solution delayed avocado white root rot (caused by *Rosellinia necatrix*) disease spread (Arjona-Girona et al., 2014), while foliar spray or drenching with 6-PP inhibited the development of disease symptoms caused by *Uncinula necator* (Pascale et al., 2017). Applying 6-PP solution via foliar spray or drenching in greenhouse or field experiments has improved crop yield and increased levels of polyphenols and antioxidant activity in grapes (Pascale et al., 2017). These findings suggest that 6-PP may play a role in inducing plant systemic resistance, as evidenced by reduced disease symptoms and the upregulation of defense genes (**Figure 4B**).

El-Hasan and Buchenauer (2009) found that infection of maize plants by *F. moniliforme* resulted in a significant slowdown in coleoptile growth and dry weight increase. However, when a 6-PP soil drench was applied four days before inoculation with *F. moniliforme*, notable disease suppression was observed, often accompanied by plant growth promotion compared to untreated and inoculated control groups. This protective effect correlated with significantly increased activities of peroxidase (PO), polyphenol oxidase (PPO), and β-1,3-glucanase in shoot and root tissues of plants treated with 6-PP compared to untreated controls. These findings suggest the potential of 6-PP to combat seedling blight disease caused by *F. moniliforme* under greenhouse conditions by activating defense mechanisms in maize plants against the pathogen (**Figure 4B**).

6-PP, synthesized by *T. atroviride*, promotes the growth of tomato and canola seedlings while also triggering plant systemic defense mechanisms against *B. cinerea* and *Leptosphaeria maculans* (Vinale et al., 2008). In addition, the upregulation of PR encoding proteins was detected in the 6-PP treated plants. While PR-1 was induced in canola, an endochitinase was induced in tomato (Vinale et al., 2008). Interestingly, using 10 mg/L of 6-PP did not show significant differences in protection against *L. maculans*, while using 1 mg/L resulted in considerable protection compared to untreated plants. Additionally, PR1–1 defense gene expression was induced by 6-PP. These results suggest that 6-PP may regulate plant growth and activate plant defense responses, with optimal effectiveness observed at lower concentrations (Vinale et al., 2008). Seed treatment with *Trichoderma* strongly induced the endochitinase IV but 6-PP did not, indicating other metabolites or mechanisms might be involved in the expression (Vinale et al., 2008). Moreover, the levels of lycopene, an important antioxidant present in tomatoes, increased by 52% after exposure to a biopolymer combined with 6-PP, isolated from *T. harzianum*, compared to the untreated samples (Carillo et al., 2020) (**Figure 4B**).

### 5.5 6-PP induces reactive oxygen species and plant defense genes in leaves

In a hydroponic system, supplementing the nutrient solution with 6-PP induced the accumulation of H_2_O_2_ and enhanced the activity of ROS-scavenging enzymes, including catalase, peroxidase, and superoxide dismutase, in tomato leaves (Hao et al., 2023). Moreover, 6-PP was observed to stimulate the transcription of NPR1 which subsequently led to the activation and accumulation of salicylic acid signature genes (PR1, PR2 & PR5). This suggests that the expression of genes associated with disease resistance was triggered by 6-PP, ultimately leading to improved disease resistance in tomatoes (Hao et al., 2023) (**Figure 4B**).

In tobacco plants, manual spraying of 6-PP extracted from *T. koningii* leads to elevated levels of proline, serving as a non-enzymatic antioxidant, and enhances the activity of pathogenesis-related enzymes such as superoxide dismutase, peroxidase, and polyphenol oxidase. These effects suggest that 6-PP functions as an elicitor, triggering resistance induction against *Tobacco mosaic virus*. At the molecular level, treatment with 6-PP resulted in accelerated and heightened expression of defense-related genes, including PR-a, PR-b, and PR-10 (Taha et al., 2021) (**Figure 4B**).

## 6 6-PP agricultural applications and ecological implications

**Figure 5:**
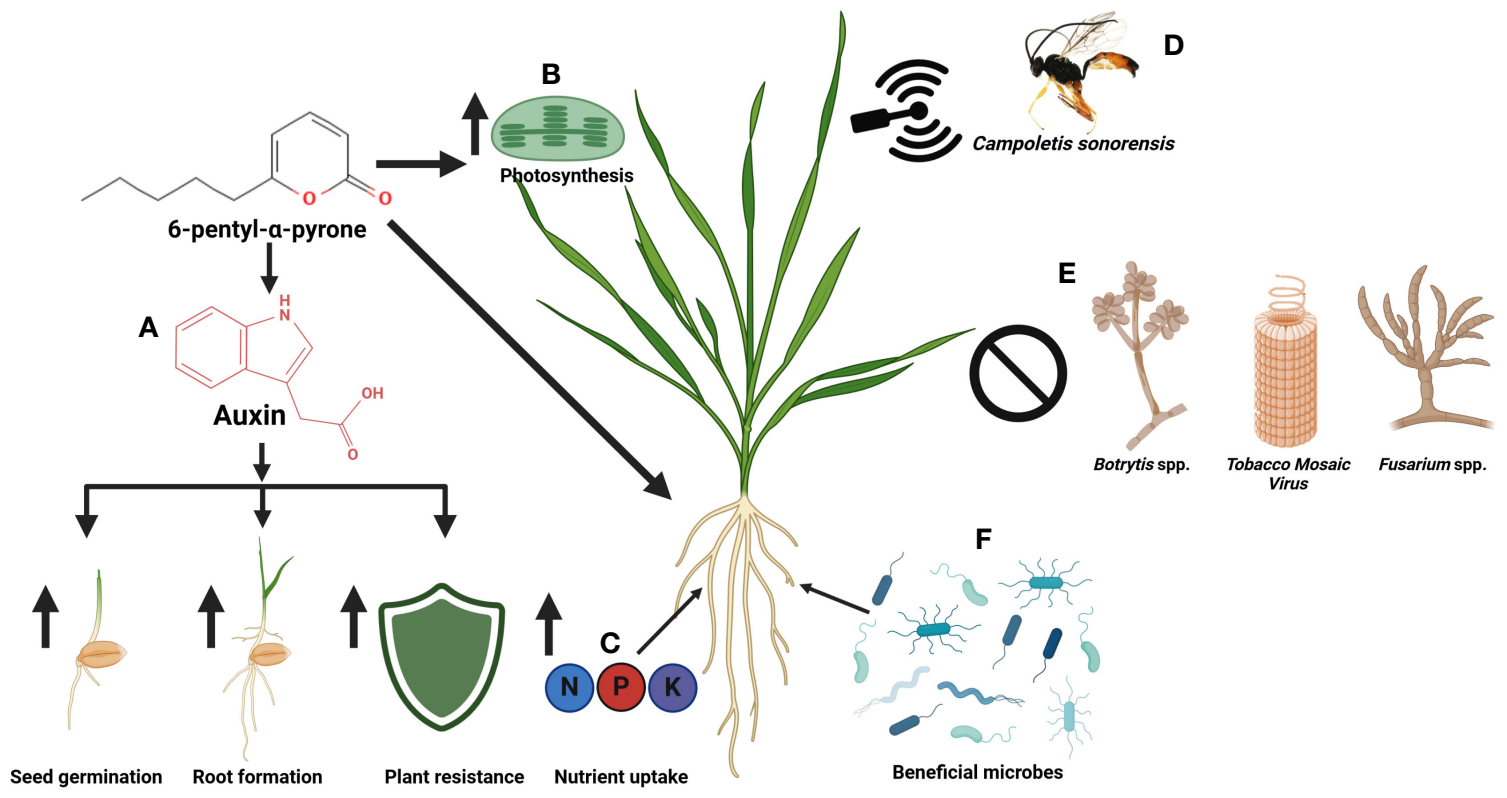
Beneficial applications of 6-penty-alpha-pyrone in agriculture **(A)** 6-PP regulates auxin for seed germination, root formation, and enhanced plant resilience to abiotic stresses (Garnica‐Vergara et al., 2016), **(B)** 6-PP improves photosynthesis activity for plant growth (Comite et al., 2021), **(C)** 6-PP enhances plant nutrient absorption (Carillo et al., 2020), **(D)** 6-PP acts as an infochemical communication method to attract a beneficial wasp (Contreras-Cornejo et al., 2018), **(E)** 6-PP helps control plant pathogens (Kottb et al., 2015; Taha et al., 2021; Hao et al., 2023), and **(F)** 6-PP induces plant signal to stimulate beneficial microbiomes (Zhu et al., 2023).

6-PP can act positively on plant health in multiple ways (**Figure 5**). This organic compound uses mechanisms such as the promotion of root growth through modulation of auxin signaling, as well as increasing tolerance in stressed plants through the activation of stress-related genes and antioxidant systems, all of which contribute to a high level of plant resilience (Garnica-Vergara et al., 2016; Cruz-Magalhães et al., 2019; Carillo et al., 2020). Additionally, it positively affects photosynthesis effectiveness, improves nutrient uptake, and possibly functions as a signaling molecule or coordinates various molecular events in the plant cell (Vinale et al., 2012; Hamrouni et al., 2020; Comite et al., 2021). A positive correlation between the ability to produce the compound and the biocontrol potential of fungal isolates has been previously reported (Degani et al., 2021). Interestingly, 6-PP induces soil structure-related processes like the establishment of soil microbial communities, which are important for plant-microbe interactions in the rhizosphere and for soil nutrient circulation (Macías-Rodríguez et al., 2020). While the molecular mechanisms underlying this beneficial impact remain speculative, these diverse pathways indicate the advantage of 6-PP as an efficient tool for plant growth promotion.

6-PP has, as yet, only been used in a limited number of glasshouse and net-house experiments to explore its impact on various biological systems (**Table 3**). 6-PP, when applied in a seed coating, as a soil drench, or as a foliar spray, was able to suppress pathogens (**Table 3**). In maize and grapes, a significant increase in crop yield resulting from pathogen control was reported (Pascale et al., 2017; Degani and Gordani, 2022). There is a need for many more field and glasshouse experiments with 6-PP to better understand its biological activities and ecological implications. These studies will be essential for elucidating the potential risks and benefits associated with 6-PP exposure in natural and agricultural ecosystems.

**Table 3:** Effect of 6-PP on plant pathogens and host plants.

Crop/Pathogen (Disease)	Method of application/dosage	Result	Reference
Maize/*Magnaporthiopsis maydis* (late wilt)	Seed coat (30µg/seed)	94–98% decrease in infection 90–120% increase in plant biomass 60% increase in cob weight	Degani and Gordani (2022)
Maize/*F. moniliforme* (seedling blight)	Soil drench (200mg/l)	Suppressed seedling blight and promoted plant growth	El-Hasan and Buchenauer (2009)
Tobacco/*Tobacco mosaic virus*	Detached leaf assay (10–30µg/ml)	Symptoms inhibited by 10–60%	Taha et al. (2021)
	Detached leaf assay (40–50µg/ml)	Symptoms inhibited by 100%	
*Arabidopsis*/*B. cinerea*	Seedling application in soil (2mM)	Significant reduction in symptoms	Kottb et al. (2015)
*Arabidopsis*/*Alternaria brassicicola*	Seedling application in soil (2mM)	Significant reduction in symptoms	
Litchi/*Peronophythora litchii* (downy blight)	Fumigation	Inhibited pathogen growth & sporangial germination; improved fruit storage	Xing et al. (2023)
Grapes/*Uncinula necator* (powdery mildew)	Foliar spray (1 & 10µM) Soil drenching (1µM)	Disease suppression and improved crop yield; increased polyphenols and antioxidant activity	Pascale et al. (2017)
Lentils/*Athelia rolfsii* (root rot)	Soil application (1–10mg/ml of ethanol)	Disease suppression	Dodd et al. (2000)

6-PP has also been used in post-harvest treatment to protect fruit from spoilage pathogens. Metabolome analysis revealed decreased lipids and increased organic acids in litchi fruits following 6-PP treatment, suggesting potential benefits in mitigating disease effects and extending shelf life. Under *P. litchii* infection conditions and during fruit storage, 6-PP altered the metabolite profile of litchi fruit. It stimulated amino acid metabolism and enhanced the tricarboxylic acid (TCA) cycle, potentially bolstering disease resistance against litchi downy blight and prolonging fruit shelf life (Xing et al., 2023). Applying 6-PP to artificially inoculated litchi fruits effectively extended shelf life and preserved freshness by boosting antioxidant activity and suppressing the disease. The abundance of 13 organic acids and derivatives was also increased, including four amino acids: L-asparagine, L-proline, L-tyrosine, and L-histidine (Xing et al., 2023). Lombardi et al. (2020) reported that 6-PP did not significantly affect the nutritional value of strawberry but did increase the quantity of specific proteins that regulate metabolites. Moreover, the tomatoes’ fruit metabolites are increased without compromising the fruit quality (Carrillo et al., 2020).

Besides its role in plant health and protection, 6-PP also improves plant product quality (Carillo et al., 2020). 6-PP has long been used as a natural flavoring agent in the food industry due to its distinct coconut scent (Oser and Ford, 1978) and is considered safe for human consumption. The European Parliament, in approving flavoring substances according to the European Union Food Improvement Agents (EUFIA) safety assessment, included 6-PP in Regulation No872/2012 (EU Food Improvement Agents, 2012). It has also been used in post-harvest treatment against *B. cinerea* in kiwifruit (Poole et al., 1998) and blueberry (Bello et al., 2022). Additionally, as several fruits, including peach, produce 6-PP, the compound could be considered biodegradable and environmentally friendly (Eduardo et al., 2010).

## 7 Challenges and future directions

The ever-increasing human population continues to threaten future global food security (Bouwmeester et al., 2019). Biopesticides based on 6-PP, a natural fungal volatile compound, could provide an eco-friendly, sustainable management method for future agricultural production, and to help combat the growing biotic stress conditions caused by climate change. Like other natural products, 6-PP is biodegradable without harming the environment (Nieto-Jacobo et al., 2017; Yao et al., 2023). Therefore, long-term consequences to environmental health from using 6-PP are unlikely to be of concern. However, how to use 6-PP in agriculture in a practical and efficient manner requires well-thought-out strategies.

The potential development of resistance in fungi could pose a limitation to the successful application of 6-PP (Walter et al., 2000). This resistance may arise directly from utilizing the metabolite or bioconversion into new molecules with reduced activity. Diverse organisms, including plants, fungi, and bacteria, can metabolize 6-PP into less toxic compounds through various processes such as oxidation, reduction, hydroxylation, and conjugation as part of their strategy to eliminate the metabolite which would otherwise be toxic (Poole and Whitaker, 1997; Cooney et al., 1997b; Cooney and Lauren, 1999; Cooney et al., 2000). These transformations often result in forming derivatives with altered chemical structures and properties (Poole and Whitaker, 1997; Cooney et al., 2000).

**Figure 6:**
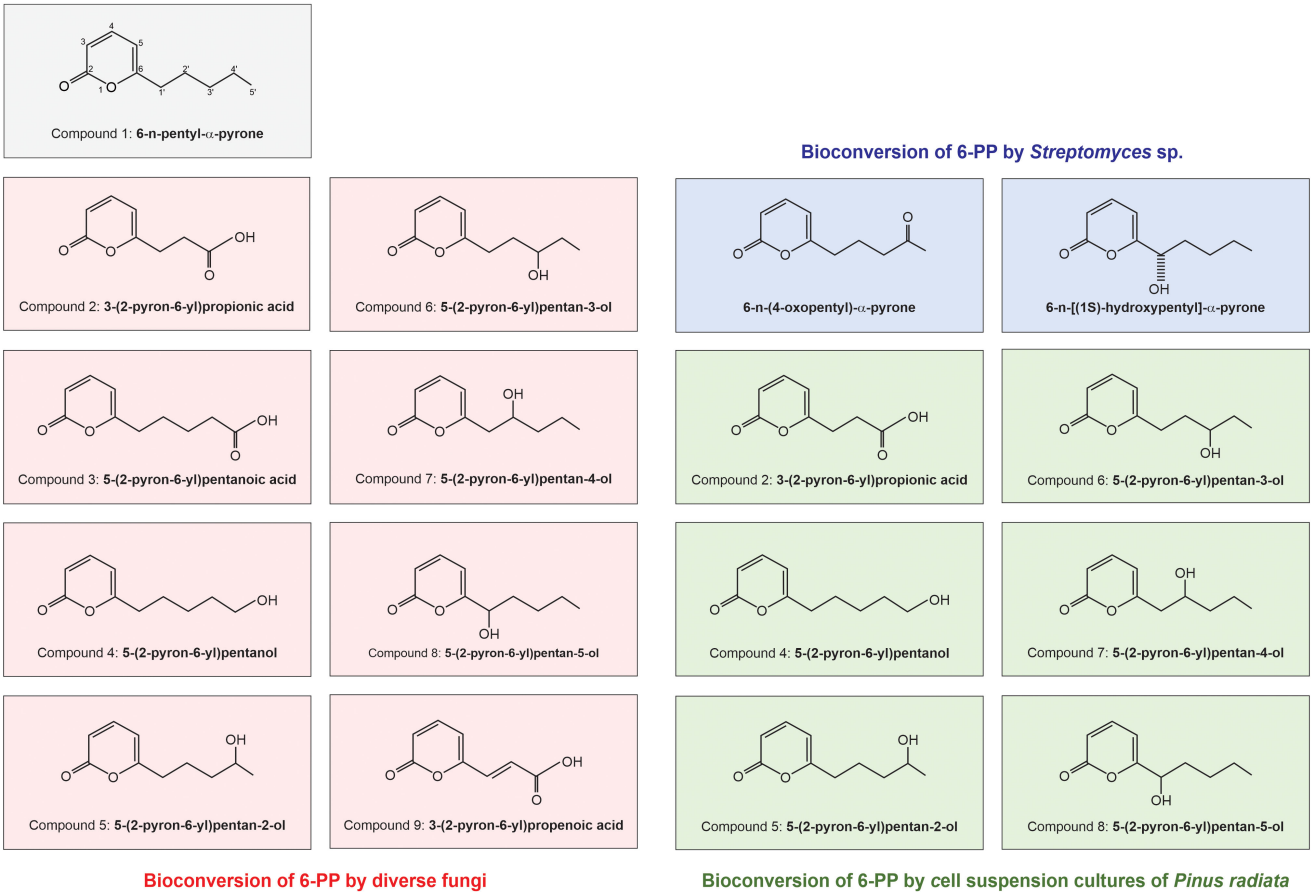
Representation of bioconversion of 6-n-pentyl-α-pyrone (6-PP) by diverse organisms. In the top panel, the structure of 6-PP is depicted. This figure illustrates the diverse bioconversion capabilities of different organisms when exposed to 6-PP, resulting in the production of various converted molecules. These conversion products are represented by different molecules derived from 6-PP, indicated within colored squares: 6-PP converted by different fungi is represented in red (Cooney et al., 1997b; Cooney and Lauren, 1999). 6-PP converted by cell culture pine is depicted in green (Cooney et al., 2000). 6-PP converted by *Streptomyces* is shown in blue (Li et al., 2005).

The oxidation of 6-PP (compound 1, **Figure 6**) by various fungi results in the formation of hydroxylated metabolites at different positions on the pentyl side chain. This detoxification mechanism appears widespread among fungal genera, indicating a common ability to oxidize compounds through hydroxylation. Different plant pathogens biotransform 6-PP, and at least 11 derivatives have been identified (See **Figure 6**). Depending on the fungus, oxidation through monohydroxylation occurred at different positions along the pentyl side chain of 6-PP. *B. cinerea* transforms 6-PP by an extracellular oxidation process, producing at least three metabolites oxygenated on the aliphatic chain: compound 2: 3-(2-pyron-6-yl) propanoic acid, compound 4: 5-(2-pyron-6-yl) pentanoic acid and either compound 5: 5-(2-pyron-6-yl) pentanol or 5-(2-pyron-6-yl) pentan-2-ol, rather than hydrolytic cleavage of the pyrone ring (**Figure 6**) (Poole and Whitaker, 1997; Cooney et al., 1997b; Cooney and Lauren, 1999). Compounds 8 and 5 were more prevalent than compounds 4, 7, or 6. However, in *B. cinerea*, the nitrogen levels in the media alter the relative abundance of these metabolites. Additionally, *B. cinerea* and several *Penicillium* and *Sclerotinia* isolates showed significant oxidation to carboxylic acid compounds 2 and 3. For the *F. crookwellense* isolate, compound 3 was present in small quantities, while compound 2 was not detected (Cooney and Lauren, 1999). Even though the efficacy of 6-PP may be degraded over time, its usage avoids the problems associated with introducing live organisms, which can encounter stringent regulations for commercialization on a large scale (Harman et al., 2010).

Compound 9, an unsaturated acid, was produced in minor amounts by the *Penicillium* isolates and *S. sclerotiorum*. This production of hydroxylated metabolites is believed to be part of a fungal strategy to neutralize potentially harmful compounds. Previous tests on the biological activity of compound 1 (6-PP) and its transformation to compounds 2 to 5 revealed reduced toxicity compared to 6-PP in fungal germination assays (Cooney and Lauren, 1999). Interestingly, cell suspension cultures of *P. radiata* modify 6-PP via hydroxylation of the pentyl side chain, producing similar derivatives of the 6-PP produced by fungal pathogens (Cooney et al., 2000), while the incubation of 6-PP in the presence of *Streptomyces* sp. induced the conversion of this molecule into two less active molecules (Li et al., 2005) (**Figure 6**). This suggests that resistance or efficacy loss may be possible. The co-application of *Trichoderma* strains with 6-PP may provide an alternative, because of the production of other fungal bioactive compounds that directly or indirectly benefit plants.

Other challenges include the cost of quantitative field experimentation required to determine the best application method, effective application rates for various crops and pathogens, and the longevity of the 6-PP priming effect. The application of 6-PP and the rate could be changed depending on the application’s purpose and method. For example, spraying 1 ppm to roots or 1µM to shoots induced an auxin-like growth effect on wheat and tomato, while for pathogen control, a higher rate might be needed (Degani et al., 2021).

Research on eco-friendly formulations or carriers as a mode of delivery is needed to minimize any possible impact on non-target organisms (Schmaltz et al., 2023). Modification of the 6-PP structure and utilization of complementary natural additives (e.g. nano-cellulose, Squire et al., 2024) may be useful for optimizing the priming response of 6-PP to plant pathogen stress. Advanced technologies like drone applications, sensor spraying, and product nanotechnology (Vanloqueren and Baret, 2008) may save resources (Sishodia et al., 2020), particularly the 6-PP component in foliar sprays. Amalgamating the 6-PP compound into Integrated Pest Management systems (Stenberg, 2017) offers another mechanism to maximize the suppression of pathogens. A holistic approach by engaging researchers, agronomists, environmentalists, and social scientists to endorse safe guidelines or policies (Siebrecht, 2020) plays a significant role in the efficient, safe, and publicly accepted use of 6-PP.

Exploring synergistic interactions between 6-PP and other biostimulants and biocontrol agents may enhance the efficacy of 6-PP for disease management. For example, Carillo et al. (2020) applied mixtures of 6-PP, *Trichoderma*, phytohormones, plant extracts and a biopolymer (a carboxymethyl cellulose) to plum tomatoes and found biocontrol and plant growth-promotion effects.

Advanced techniques such as genomics, metabolomics, proteomics, and transcriptomics are beginning to be associated with plant production data using novel tools such as network analysis, large-scale data integration and machine learning approaches (Bouwmeester et al., 2019). This will facilitate the interpretation of the biological activity of VOCs defense in various plant physiological and biochemical mechanisms. With these advanced approaches, the discovery of biosynthetic gene clusters will also further accelerate and allow better understanding of the role of VOCs in the plant and organism interactions (Bouwmeester et al., 2019).

Addressing the challenges discussed above and pursuing future directions will be crucial to harness the potential of 6-PP as an agent to prime plant defense mechanisms providing direct and indirect control of plant pathogens and promoting growth, ultimately contributing to sustainable crop production and food security (Comite et al., 2021). Cost-effective production, effective application methods, and market acceptance will also be critical for the commercial success of 6-PP based products.

## 8 Conclusions

Using 6-PP for disease control involves a multifaceted action including intricate cellular and molecular interactions. These include disruption of cell membranes, inhibition of critical enzymes, induction of oxidative stress, and modulation of gene expression. These mechanisms collectively underscore 6-PP’s antimicrobial efficacy and potential as a biopesticide for managing plant diseases. However, further research is imperative to unravel the precise molecular targets and signaling pathways implicated in 6-PP-mediated inhibition of pathogen growth and virulence.

Moreover, the capacity of 6-PP to induce plant defense responses in systemic tissues offers promising avenues for strategic application, whether through soil drenching or seed treatment, ensuring protection for tissues distant from the application site.

Advancing our understanding of the intricate interplay between plants and 6-PP in the presence of pathogens is promising for refining biopesticide applications in agriculture. Growers stand to benefit from optimizing 6-PP utilization through eco-friendly carriers and precision agriculture techniques, seamlessly integrating them into Integrated Pest Management systems. This strategic approach will help address the challenges of large-scale commercialization of novel products and foster the transition towards sustainable agricultural practices (Campuzano et al., 2023).

## Author contributions

AM-M: Conceptualization, Data curation, Formal analysis, Funding acquisition, Investigation, Project administration, Resources, Supervision, Validation, Visualization, Writing – original draft, Writing – review & editing. EE-N: Conceptualization, Data curation, Formal analysis, Investigation, Validation, Visualization, Writing – original draft, Writing – review & editing. SS: Data curation, Investigation, Visualization, Writing – review & editing, Writing – original draft. HW: Conceptualization, Data curation, Investigation, Writing – review & editing, Writing – original draft. HA: Data curation, Investigation, Supervision, Writing – review & editing, Writing – original draft. JE-A: Data curation, Formal analysis, Investigation, Validation, Writing – review & editing, Writing – original draft. DK: Data curation, Formal analysis, Investigation, Visualization, Writing – review & editing. JH: Data curation, Formal analysis, Funding acquisition, Investigation, Supervision, Validation, Writing – review & editing, Writing – original draft.
